# Mitochondrial Unfolded Protein Response Under Hypoxic Conditions

**DOI:** 10.7759/cureus.117584

**Published:** 2026-10-07

**Authors:** Gamze Bilgili, Fevzi Sökmen, Yunus Emre Cavlak, Hasan H Kazan, Ahmet Caglar Ozketen

**Affiliations:** 1 Department of Medical Biology, Faculty of Health Sciences, Başkent University, Ankara, TUR; 2 Department of General Internal Medicine, Gülhane Faculty of Medicine, University of Health Sciences, Ankara, TUR; 3 Department of Medical Biology and Genetics, Faculty of Science and Letters, Başkent University, Ankara, TUR; 4 Department of Medical Biology, University of Health Sciences, Ankara, TUR; 5 Department of Molecular Medicine, DESAM Research Institute, Near East University, Nicosia, TUR

**Keywords:** hypoxia, hypoxic pathology, mitochondria, proteostasis, unfolded protein response

## Abstract

Hypoxia is a critical condition affecting the physiological and pathophysiological processes in the cellular environment due to depleted oxygen levels. Under hypoxic conditions, cells undergo a series of changes to adapt to the lack of oxygen that is vital for cellular metabolism in higher organisms. Importantly, hypoxia may induce organelle-specific molecular alterations, including misfolded/unfolded proteins. As a fundamental response to these changes, the unfolded protein response (UPR) is triggered by organelles, including the endoplasmic reticulum (ER) and mitochondria. UPR in mitochondria (UPRmt) is a mitochondrial stress-adaptation pathway that communicates mitochondrial dysfunction to the nucleus to induce response genes such as chaperones and proteases, distinct from UPR in the endoplasmic reticulum (UPRer).

Although UPR in the ER under hypoxic conditions has been well documented, the association of UPRmt has not been clearly evaluated. Given the fact that mitochondria are one of the pivotal organelles where oxygen consumption carries utmost importance, understanding the underlying mechanisms is crucial to overcoming hypoxic pathology and maintaining mitochondrial homeostasis.

In this study, we reviewed the literature to dissect studies combining UPRmt under hypoxia in various physiological and pathophysiological conditions, including the regulation of tissue barrier homeostasis in diverse organisms, to present a comprehensive perspective. Accumulatively, the studies have revealed the protective effects of UPRmt under hypoxic conditions; yet some of them have reported that the association between hypoxia and UPRmt could be organism-specific. Even so, the number of studies is limited to systematically conclude the involvement of UPRmt in hypoxia. Therefore, further molecular studies are still needed.

## Introduction and background

Molecular oxygen is a vital molecule for aerobic organisms to maintain and regulate cellular processes. It is consumed in adenosine triphosphate (ATP) production as the final electron acceptor. The absence or deficiency of available oxygen in the cellular environment is called hypoxia, and it is a critical factor affecting both physiological and pathological conditions. Although oxygen is inevitably required for cellular functions, cells can adapt to hypoxic conditions by fine-tuning transcriptional and post-transcriptional regulation. Due to their involvement in multiple pathophysiological conditions, including pathogen infections, cancer progression, and other diseases, hypoxic pathways are among the most studied mechanisms [1].

Hypoxia-inducible factors (HIFs) are heterodimeric protein complexes having α and β subunits that function as central regulators of the cellular response to reduced oxygen availability [1,2]. The α subunit is hydroxylated on prolyl residues by the oxygen-dependent prolyl hydroxylase domain (PHD) family and is subsequently targeted for proteasomal degradation. This degradation is inhibited under hypoxic conditions, leading to interaction between the α and β subunits. Both the β subunit and HIF-1α are constitutively expressed in body tissues, whereas the close paralog HIF-2α and the divergent paralog HIF-3α are expressed in specific tissues and may differ in their biological functions [1]. The correlation between hypoxia and several phenotypes, including heart, pulmonary, liver, and kidney diseases, inflammation, neurodegeneration, and cancer, with respect to their molecular mechanisms, has been extensively documented in the literature [3-7].

Hypoxia has a decisive role, particularly in mitochondrial function. Mitochondria, so-called the powerhouse of the cell, are membrane-bound organelles derived from prokaryotes and the center of ATP production by the respiratory chain in eukaryotic cells. They are critically essential for energy homeostasis, signaling, stress responses, and cellular death mechanisms [8]. Hypoxia causes decreased production of metabolites such as acetyl-CoA and aspartate by negatively affecting the tricarboxylic acid (TCA) cycle in mitochondria. Through the activities of HIF1 and HIF1-dependent proteins, energy homeostasis is maintained by the modulation of the electron transport chain (ETC) under short-term hypoxic stress, while ETC assembly is blocked to reduce reactive oxygen species (ROS) under long-term hypoxic stress. ROS levels are increased in some circumstances, such as during the inhibition of mitochondrial function under hypoxia [1,3]. Moreover, calcium levels are elevated under hypoxia due to effects on membrane channels, which limits ATP consumption. Besides cellular metabolism, hypoxia could also affect other cellular dynamics, including protein folding [7].

In addition to the cellular effects, hypoxia could reorganize tissue barriers. Under hypoxic conditions, tight junctions, which are the structural components of tissue barriers, would lose their integrity. Here, the main regulator of hypoxia, HIF1, can control the expression of claudins, which are integral membrane proteins supporting the formation of tight junctions to prevent loss of tissue barrier integrity [9,10]. The function of HIF1 under hypoxia in terms of tissue barrier regulation has frequently been demonstrated in the intestinal tract. HIF1 targets mucin-coding genes, whose protein products inhibit the direct interaction between mucosal and luminal contents, which is fundamental for the function of the intestinal epithelium [11]. Moreover, HIF1 is able to control the expression of intestinal trefoil factor-3, regulating the migration of intestinal epithelial cells and promoting protection of the epithelial barrier [12]. Contrary to these positive effects, under hypoxia, using in vivo rat models, HIF1 has been shown to cause disruptions in the blood-brain barrier (BBB) and increase BBB permeability through the upregulation of vascular endothelial growth factor (VEGF) [13]. These reports underline a dual role of HIF1 in the regulation of barrier integrity in a tissue-specific or context-dependent manner. Concisely, HIF activation may contribute to protective metabolic adaptation, epithelial survival, barrier maintenance, and angiogenic adaptation. In contrast, it may also contribute to barrier dysfunction, excessive VEGF signaling, vascular permeability, inflammatory signaling, and endothelial dysfunction under certain pathological conditions.

Protein misfolding and aggregation are crucial stress conditions linked to numerous pathological conditions [14]. Although it has been extensively studied in the cytoplasm, protein misfolding and aggregation in other cellular compartments, especially in mitochondria, have also been emphasized to result in pathological conditions [15]. Several studies on mitochondria have shown that stress conditions such as ageing and hypoxia could increase the amounts of insoluble proteins, pointing out misfolded or aggregated proteins that activate the unfolded protein response (UPR) pathway [15-17].

Recently, Zhou et al. reviewed the involvement of hypoxia in UPR, notably in endometriosis, a chronic gynecological disease, by focusing on endoplasmic reticulum (ER) stress [18]. Although UPR mechanisms in the ER and mitochondria crosstalk to initiate the integrated stress response (ISR), the UPR in mitochondria (UPRmt) under hypoxia should be evaluated separately owing to the unique properties of mitochondria in both cellular metabolism and cell death. Thus, the present review article aims to combine all studies concerning UPRmt under hypoxic conditions. We briefly summarize the UPRmt mechanisms and present research articles with a focus on the UPRmt-hypoxia axis.

## Review

Mitochondrial UPR

UPRmt is a transcriptional response occurring via communication between the mitochondria and the nucleus. UPRmt is initiated as a result of the accumulation of unfolded or misfolded proteins within the mitochondrial matrix. It was first defined by a report in which chaperones and proteases were shown to be synthesized from the nuclear genome in the presence of unfolded mitochondrial proteins. Cumulatively, unfolded protein levels in the mitochondria, the presence of any damage to mitochondrial DNA, and irregularities in oxidative phosphorylation (OXPHOS) activity have been shown to trigger UPRmt [19].

The mechanisms of UPRmt have frequently been studied in *Caenorhabditis elegans* (*C. elegans*) [20]. Findings from *C. elegans* regarding UPRmt have further been adapted to mammals to decipher the possible players involved in UPRmt. Accordingly, the mechanistic pathway of UPRmt in mammals has been clearly identified using different cell or animal models that mimic diverse phenotypes, such as cancer, aging, and other pathophysiological conditions, including fatty liver disease [21-23].

UPRmt requires the regulation of gene expression and chromatin remodeling within cells through epigenetic modifications or between tissues through mitokines [19]. Any signal of mitochondrial damage, including ROS, decreasing amounts of amino acids, ER stress, and mitochondrial unfolded proteins, causes the phosphorylation of eukaryotic translation initiation factor 2α (eIF2α) by general control nonderepressible 2 (GCN2) and protein kinase R (PKR)-like endoplasmic reticulum kinase (PERK). eIF2α phosphorylation suppresses global translation initiation by reducing ternary complex availability. This restriction triggers the selective translation of stress-responsive transcripts, such as the CCAAT/enhancer-binding protein (C/EBP) homologous protein (CHOP), activating transcription factor 4 (ATF4), and activating transcription factor 5 (ATF5), thereby driving cellular transcriptional reprogramming to cope with stress [24,25]. These proteins, specifically ATF5, are responsible for the production of mitochondrial chaperones and proteases to eliminate mitochondrial damage [24,25]. Under normal conditions, ATF5, containing both a mitochondrial targeting sequence (MTS) and a nuclear localization signal (NLS), is internalized into the mitochondria by the translocase of the inner membrane (TIM) and translocase of the outer membrane (TOM) complexes and then degraded. However, if any damage to the mitochondria occurs, ATF5 cannot be internalized into the mitochondria; rather, it goes to the nucleus owing to the NLS and regulates the expression of chaperones and proteases, which are critical for repairing the damage [23].

Activating transcription factor associated with stress 1 (ATFS-1) of *C. elegans*, homologous to mammalian ATF5, functions as the central transcriptional regulator of UPRmt by sensing mitochondrial import efficiency and shuttling between cellular compartments. Under basal homeostasis, ATFS-1 is imported into mitochondria via its N-terminal MTS and rapidly degraded by the Lon protease [26,27]. However, compromised membrane potential impedes ATFS-1 import, allowing its accumulation in the cytosol and translocation to the nucleus via a C-terminal leucine zipper-based NLS. In the nucleus, ATFS-1 binds specific promoter sequences to upregulate mitochondrial chaperones, proteases, antioxidant systems, and metabolic adaptation genes [19].

UPRmt could also occur through chromatin remodeling, which has been well documented in *C. elegans*. The activation of UPRmt also requires chromatin reorganization, which is dependent on the histone methyltransferase 2 (MET-2) and nuclear cofactor LIN-65. Normally, LIN-65 is located in the cytoplasm, while the homeobox domain transcription factor (DVE-1) and ubiquitin-like protein (UBL-5) are located in the perinuclear region. The activation of MET-2 occurs during mitochondrial stress and directs LIN-65 to the nucleus for chromatin remodeling [19,27]. Subsequently, ATFS-1, DVE-1, and UBL-5 interact with the promoters of UPRmt-responsive genes, such as mitochondrial chaperones and proteases. Histone lysine demethylases JMJD-1.2 and JMJD-3.1 have also been demonstrated to be UPRmt regulators through chromatin remodeling [26]. Consequently, chromatin remodeling is required to induce UPRmt-responsive genes. 

Four different pathways of UPRmt have been reported so far and well reviewed by Münch (2018) [28]. These pathways are the canonical, sirtuin, estrogen receptor-α (ERα), and translation axes (Figure 1). (i) The canonical axis is one in which ATF5 has a role in the transcriptional stress response activated by the accumulation of misfolded proteins, as demonstrated by in vivo studies [29]. (ii) In the sirtuin axis, sirtuin 3 (Sirt3), which is a member of the sirtuin family of lysine deacylases, deacetylates Forkhead box O3A (FOXO3A), resulting in the localization of FOXO3A to the nucleus. In the nucleus, FOXO3A increases the expression of antioxidant proteins, including superoxide dismutase 2 (SOD2) and catalases. Accordingly, the overexpression of Sirt3 triggers UPRmt through the Foxo3a/Sphk1 pathway, resulting in mitochondrial protection in neurons of a transgenic mouse model [30]. (iii) In the ERα axis, ERα is activated by AKT serine/threonine kinase, which is activated by mitochondrial unfolded proteins. Activated ERα increases the expression of nuclear respiratory factor 1 (NRF1), which further enhances the expression of specific proteases, such as high-temperature requirement serine protease A2 (HtrA2/Omi), and ubiquitin-proteasome system (UPS) subunits [28,31]. Moreover, activation of the ERα axis of UPRmt in a sex-dependent manner was reported in G93A-SOD1 mouse models, which serve as an in vivo validation of ERα-mediated UPRmt [31]. (iv) Finally, in the translation axis, although it is not a fully elucidated mechanism, the pre-RNA synthesized from mitochondrial DNA cannot be processed by the mitochondrial pre-RNA processing nuclease mitochondrial ribonuclease P protein 3 (MRPP3), preventing the translation of mitochondrial proteins [28,32]. Münch and Harper (2016) revealed that UPRmt decreases the synthesis of matrix-localized proteins by means of translational inhibition upon inhibitor-mediated UPRmt induction in the human cervical cancer cell line HeLa, signifying the presence of a translational axis in UPRmt similar to that in *C. elegans* [32].

**Figure 1:**
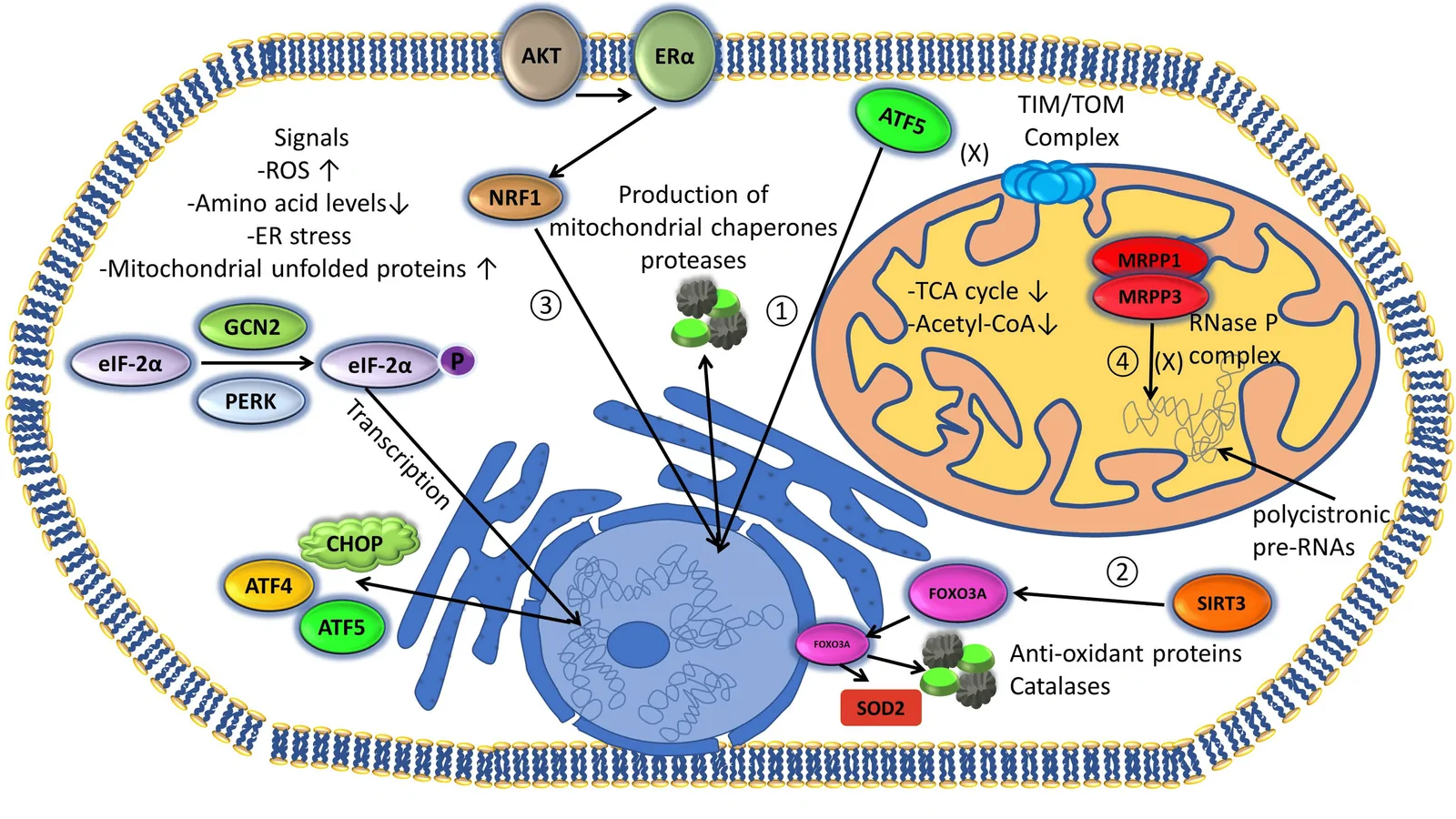
Schematic representation of four different pathways of unfolded protein response in mitochondria (UPRmt). The signals of mitochondrial damage trigger eIF2α phosphorylation, which leads to the production of several proteins, including CHOP, ATF4, and ATF5. (1) Under normoxia, ATF5 translocates into the mitochondria through the TIM/TOM complex and is degraded. Hypoxic conditions alter ATF5 localization from the mitochondria to the nucleus, triggering the production of mitochondrial chaperones and proteases. (2) SIRT3 deacetylates FOXO3A, facilitating its nuclear localization and the production of SOD2, antioxidant proteins, and catalases. (3) Activation of NRF1 by AKT and ERα results in protease expression. (4) Transcription of mtDNA generates polycistronic pre-RNAs, which are processed by the RNase P complex (MRPP1-3). Hypoxia diminishes MRPP3 levels, thereby impairing mitochondrial translation. Image credit: This is an original author-created schematic using Microsoft PowerPoint (Microsoft Corp., Redmond, WA, USA). eIF2α: eukaryotic initiation factor 2 alpha; CHOP: C/EBP homologous protein; ATF4: activating transcription factor 4; ATF5: activating transcription factor 5; TIM: translocase of the inner mitochondrial membrane; TOM: translocase of the outer mitochondrial membrane; SIRT3: sirtuin 3; FOXO3A: forkhead box O3; SOD2: superoxide dismutase 2; NRF1: nuclear respiratory factor 1; AKT: protein kinase B; ERα: estrogen receptor alpha; mtDNA: mitochondrial DNA

UPRmt has also been shown to occur systemically. In *C. elegans*, UPRmt induced by mitochondrial damage in neurons was shown to trigger the same response in the intestine through the action of FMRF-like peptide (FLP-2) and serotonin. Moreover, UPRmt was demonstrated to be a part of innate immunity in *C. elegans*, in which infection by a pathogenic microorganism was shown to correlate with UPRmt [33,34].

Mitochondrial UPR and tissue barriers

UPRmt is a crucial mechanism for preserving tissue barrier integrity by regulating mitochondrial dysfunction, responses to damage, and cell death. However, the involvement of UPRmt in tissue barrier regulation has been poorly studied in the literature regarding different types of tissue barriers, such as the intestinal barrier and BBB. Accordingly, UPRmt has been suggested to have a role in the localization and differentiation of intestinal epithelial stem cells, which are critical for the integrity of the intestinal barrier [35,36].

UPRmt functions as an architectural regulator along the crypt-villus axis by directly modulating how stem cells survive and localize within their spatial niches. A balanced UPRmt response maintains the stem cell niche within its proper anatomical zone [35]. However, upon induction of severe mitochondrial stress, such as through the deletion of the crucial mitochondrial matrix chaperone heat shock protein 60 (HSP60), UPRmt triggers a loss of normal intestinal stemness, leading to crypt structural failure and cell death. Subsequently, affected cells release WNT signaling-associated factors WNT10A and R-spondin 1 (RSPO1) through a paracrine stress mechanism [35]. This acts as a signal to nearby stem cells, driving localized hyperproliferation to restore the damaged crypt architecture.

Similarly, in inflammatory bowel disease (IBD), in which immune regulation is imbalanced owing to alterations associated with a dysregulated epithelial barrier, UPRmt has been speculated to be activated through crosstalk with the UPR in the ER [37]. In animal models of cerebral ischemia/reperfusion injury (IRI), which causes BBB disruption, activation of UPRmt by Sirt3 overexpression was shown to have a protective effect in terms of preserving mitochondrial function and neural viability [30]. Induction of UPRmt by natural agents, upregulation of ATF5 and Lon peptidase 1 (LONP1; a mitochondrial protease involved in UPRmt), and HSP60 (a mitochondrial chaperone involved in UPRmt) have been shown to be protective by stabilizing junction assembly and promoting BBB integrity [38]. These examples demonstrate the importance of UPRmt not only in cellular homeostasis but also in tissue barrier integrity.

Mitochondrial UPR under hypoxia

Extreme hypoxia could cause cell death through diverse mechanisms, particularly through disruptions in cellular energy metabolism. When oxygen is depleted, critical tissues, including neurons of the central nervous system and cardiac myocytes, are remarkably affected. Nevertheless, the possibility of recovering affected tissues after hypoxia has drawn considerable attention [39].

Mitochondria are the primary organelles that are widely influenced by hypoxia, as oxygen is the terminal electron acceptor of the mitochondrial ETC, contributing to proton pumping to generate ATP through ATP synthase. Decreased oxygen levels, however, bring about disruptions in mitochondrial proteins and ATP synthesis and elicit ROS production. Moreover, in addition to the regular functions of mitochondrial proteins, their synthesis and folding properties may also be disturbed, which points to fundamental alterations [40,41]. Hypoxia-induced UPRmt further triggers mitochondrial protein aggregation [42]. Thus, it is essential to comprehensively explore the association between UPRmt and hypoxia.

In a study conducted in *C. elegans*, hypoxia was shown to induce failure of mitochondrial protein homeostasis even at early stages, and UPRmt was protective against hypoxic injury. Importantly, the tolerance of early-stage hypoxia by UPRmt, which results in the evasion of cell death, was discussed, as essential proteins in the mitochondria may be more resistant to hypoxia, and long-term hypoxic conditions may be required to trigger cell death [42]. Following this report, the same research group mechanistically identified hypoxia-dependent mitochondrial protein aggregation. Using reporter systems with fluorescent protein-labeled insoluble mitochondrial proteins, the researchers studied mitochondrial protein aggregation during aging and hypoxia. Surprisingly, the activation of UPRmt by either aging or hypoxia stimulated increased protein aggregation in the mitochondria, which was proposed as a protective strategy. The effect of the aggregates in the mitochondria was further discussed as an opportunity to remove toxic misfolded proteins orchestrated by UPRmt [15]. 

In another study on *C. elegans*, Yan et al. (2021) questioned the possible interaction between hypoxia resistance and UPRmt. They developed hypoxia-resistant *C. elegans* models using siRNAs targeting hypoxia-specific proteins and examined the involvement of UPRmt in the resistance phenotype. According to the results, hypoxia resistance was independent of UPRmt and vice versa. However, UPRmt diminished hypoxia-induced mitochondrial protein aggregation (HIMPA), which is different from generic mitochondrial protein aggregation driven by generalized proteostasis collapse, severe oxidative damage, thermal stress, or aging. This effect of UPRmt was proposed as a preventive mechanism for early hypoxic pathologies in *C. elegans* [41], and this research was significant for exploring the molecular involvement of UPRmt in hypoxia resistance.

Mao et al. (2016) questioned the importance and role of nicotinamide mononucleotide adenylyltransferase type 1 (Nmnat1) in hypoxic injury and studied this protein in both mouse primary hippocampal neuron cultures and *C. elegans*. They demonstrated the protective role of mutant mouse Nmnat1 protein through a gain-of-function mechanism in both mouse neurons and *C. elegans* against hypoxia. Moreover, Nmnat1 protein with the gain-of-function mutation diminished mitochondrial proteostasis stress via activation of the UPRmt pathway, thereby supporting hypoxia resistance. Furthermore, increased Nmnat1 function was correlated with longevity in *C. elegans*. Finally, the mechanism of Nmnat1 was hypothesized to involve the function of its enzyme product, nicotinamide adenine dinucleotide (NAD), whose role in UPRmt had been previously reported [43].

In addition to *C. elegans* and mammals, UPRmt upon hypoxia has also been studied in other organisms such as clams and scallops. Although UPRmt was not elaborated, the effects of hypoxia and reoxygenation (H/R) on mitochondria in these organisms were comparatively investigated in the study. In both organisms, mitochondrial phosphorylation capacities were diminished to possibly store energy. However, the responses to H/R were diverse between the organisms in some aspects. Importantly, the eIF-2α, which is the initiator of the UPRmt, was not activated in scallops while being dramatically activated in clamps after H/R [44]. Hence, these results may demonstrate that activation of UPRmt could be organism-specific.

Under H/R stress, the crosstalk between mitophagy and UPRmt was elucidated in *C. elegans*. In the study, prevention of mitophagy by the loss of FUN14 domain-containing 1 (FUNDC-1), a mitophagy receptor, diminished hypoxia-dependent mitophagy under short-term exposure to hypoxia, as expected. Nevertheless, when the exposure period increased, H/R-dependent injury was unexpectedly further alleviated. As an explanation for the long-term effects of hypoxia, the involvement of ATFS-1 was considered, and UPRmt was proposed as a compensatory mechanism to alleviate H/R injury in the model organism [45]. Similar results were previously obtained in a study carried out by Peña et al. (2016). In an anoxia-based *C. elegans* model of IRI, activation of ATFS-1 was sufficient to induce UPRmt and significantly prevent death and dysfunction [46].

Even though the protective effect of UPRmt under H/R stress was widely emphasized by several studies, Xu et al. (2016) proposed a pro-apoptotic role of UPRmt under H/R stress in human endothelial cells. They demonstrated that H/R stress induced mitochondrial ROS formation and UPRmt, which further triggered apoptosis in these cells. Treatment of cells with ROS scavengers, N-acetyl cysteine or acetylcholine, blocked the formation of mitochondrial ROS and, thereafter, UPRmt. Inhibition of UPRmt was discussed as a protective mechanism against distress in mitochondrial structure and H/R [47]. Nonetheless, the effect of ROS scavengers requires additional research to determine whether apoptosis is blocked owing to inhibition of UPRmt or excessive ROS formation.

Using mouse models of defective HtrA2/Omi, a serine protease located at the mitochondrial intermembrane space, Meng et al. (2022) investigated the role of IRI, which was already shown to trigger hypoxic injury. Although the researchers did not emphasize hypoxic injury as a result of IRI, they showed that heterozygous defects in HtrA2/Omi protein in the cortex resulted in higher expression of UPRmt-related proteins via the uncoupling protein 2 (Ucp2)-Sirt3-peroxisome proliferator-activated receptor gamma coactivator 1-alpha (Pgc1) axis during IRI, in the presence of increased mitochondrial damage, pointing to UPRmt as a protective mechanism. While mice with reduced HtrA2/Omi function can temporarily maintain brain health under normal conditions via the UCP2-SIRT3-PGC1 axis, this compensation fails during IRI, leading to severe mitochondrial deficiency and larger brain infarctions. The study was mechanistically well-defined; however, the involvement of hypoxia needs further investigation [48,49].

The hypoxia-UPRmt relationship has also been studied in the pathophysiology of preeclampsia. Preeclampsia, a hypertensive disorder, is a critical reason for maternal and neonatal mortality occurring during pregnancy. In preeclampsia, placentation is defective, causing a maternal abnormal angiogenic profile [50]. The imbalance between hypoxia-mediated oxidative stress and angiogenic factors is one of the reasons for preeclampsia. Accordingly, HIF-1α, which controls oxygen homeostasis, angiogenesis, metabolism, and adaptation, has been shown to induce anti-angiogenic and block pro-angiogenic factors [51]. Yung et al. (2019) studied the involvement of UPRmt in trophoblast cells subjected to repetitive hypoxia-reoxygenation, mimicking the preeclampsia phenotype. According to the results, hypoxia-reoxygenation activated a noncanonical UPRmt via reduced caseinolytic protease proteolytic subunit (CLPP), a critical protease in UPRmt signal transduction, and increased tumorous imaginal disk 1 (TID1) chaperone. Induction of UPRmt by reduced CLPP triggered mitochondrial dysfunction and altered OXPHOS capacity. Hence, UPRmt has been proposed as a target for early-onset preeclampsia [52]. Ge et al. (2025) demonstrated that hypoxia triggers the UPRmt through a basic helix-loop-helix family member E40 (BHLHE40), a core circadian transcription factor, brain and muscle Arnt-like 1 (BMAL1), and mitochondrial fission gene (Fis1) pathway that drives mitochondrial fission and organellar dysfunction, ultimately initiating an inflammatory immune response [53]. In a recent study, Tong et al. (2025) revealed that chronic gestational hypoxia triggers a noncanonical UPRmt, characterized by increased HSP60 and TID1 chaperones without changes in ClpP [54]. Moreover, the study showed that hypoxia selectively downregulates complex I respiration to reduce oxidative stress while preserving overall placental OXPHOS capacity and inducing mitochondrial fission in sheep, signifying the relationship between mitochondrial response and chronic gestational hypoxia during pregnancy.

The studies demonstrating the relationship between hypoxia and UPRmt in various organisms were summarized in Table 1.

**Table 1:** List of studies on the relationship between hypoxia and UPRmt in the literature. ATFS-1: activating transcription factor associated with stress 1; UCP2-SIRT3-PGC1: uncoupling protein 2-Sirtuin 3-Peroxisome proliferator-activated receptor gamma coactivator 1; PERK: protein kinase R (PKR)-like endoplasmic reticulum kinase; eIF2α: eukaryotic translation initiation factor 2α; OXPHOS: oxidative phosphorylation; UPRmt: unfolded protein response in mitochondria

Molecular mechanism	UPRmt inducer/target gene	Phenotype	In vitro/in vivo models	Reference
ATFS-1 dependent	pept-1, sucl-2, C33F10.12, pdha-1, and R04F11.2	Reduced mitochondrial protein aggregation, hypoxia resistance	Caenorhabditis elegans	[41]
ATFS-1 dependent	cco-1, nduf-6, lpd-9, letm-1	Hypoxia resistance	C. elegans	[42]
ATFS-1 independent	Doxycycline, atfs-1(tm4919 and tm4525)	Hypoxia resistance	C. elegans	[42]
ATFS-1 dependent	Hypoxic stress and aging	Mitochondrial protein aggregation	C. elegans	[15]
ATFS-1 independent	m-nonN-Nmnat1	Hypoxia resistance and lifespan extension	*C. elegans*, mouse primary neurons	[43]
-	Hypoxia/reoxygenation stress	Oxidative damage protection to the membranes and susceptibility to protein damage	*Mercenaria mercenaria*, *Argopecten irradians*	[44]
ATFS-1 dependent	fndc-1	Reduced hypoxic mitophagy	C. elegans	[45]
ATFS-1 dependent	Ethidium bromide, spg-7	Protection against anoxia-reperfusion	C. elegans	[46]
-	Hypoxia/reoxygenation stress	Endothelial protection and mitochondrial protection	Human umbilical vein endothelial cells (HUVECs)	[47]
UCP2-SIRT3-PGC1 axis	HtrA2/Omi	Cellular aging and mitochondrial quality imbalances	HtrA2^mnd^2 mice models	[48]
PERK/eIF2α axis	Hypoxia/reoxygenation stress	Mitochondrial dysfunction and altered OXPHOS capacity	Trophoblast cells	[52]
-	Hypoxia	Activation of UPRmt marker genes, inflammatory immune response	in vitro (RAW264.7, bone marrow-derived macrophages) and in vivo mouse models	[53]
Non-canonical UPRmt	Chronic gestational hypoxia	Activation of non-canonical UPRmt, down-regulation of complex I respiration, induction of mitochondrial fission	Sheep	[54]

One of the most significant locations for hypoxia exposure is the tumor microenvironment due to the depletion of oxygen caused by the rapid growth of cancer cells. Understanding the relationship between hypoxia and UPRmt is critical to grasping the adaptive processes of cancer cells to resist, reprogram, and proliferate in the tumor microenvironment. Hypoxia and UPRmt associations in the tumor microenvironment have been discussed and reviewed recently by Bartoszewska et al. (2022) [55]. The UPRmt is activated by hypoxia-induced metabolic adaptation and mitochondrial proteotoxic/proteostatic stress in the cell. Modulations regulated by UPRmt are decisive in increasing survival rate and pathology [55]. The tumor microenvironment defines the ecosystem surrounding the tumor structure and consists of blood and lymphatic vessels, extracellular matrix (ECM), immune cells, and fibroblasts [56]. The relationship between the tumor microenvironment and the tumor itself could be advantageous or disadvantageous for the tumor. Cancer cells would face hypoxia when they progress rapidly because of the lack of adequate blood vessels in the tumor microenvironment [57]. Hence, exposure to hypoxic conditions causes cancer cells to induce the stabilization of HIF-α. As a cumulative response in the presence of accumulated misfolded/unfolded proteins in the ER and mitochondria, PERK signaling is activated to trigger expression of pro-angiogenic or apoptotic genes [55].

Finally, although hypoxia and UPRmt have separately been evaluated in terms of tissue barrier integrity, further molecular studies are still required to comprehensively decipher the possible additive roles of those mechanisms for diverse tissues.

## Conclusions

As a critical factor affecting both physiological and pathophysiological conditions, hypoxia remarkably adjusts the molecular mechanisms of cells for adaptation. Hypoxic conditions result in several metabolic alterations in the cell to respond to the demand for molecular oxygen. One of those adaptation strategies is UPR, which is initiated by the ER and/or mitochondria. Even though UPR in the ER has been well documented, UPRmt has not been completely investigated. The present article reviewed the studies on the association of UPRmt under hypoxic conditions in a comprehensive manner. Accordingly, the studies generally report crosstalk between HIF and ATF5 (ATFS-1, a functional homolog in *C. elegans*) to trigger UPRmt in hypoxia models. Activation of UPRmt could be beneficial or detrimental, depending on the organism and hypoxia model. Importantly, as a major entity influenced by hypoxia, regulation of tissue barrier integrity by the combination of hypoxia and UPRmt is missing in the literature, even though they have been investigated individually.

Moreover, studies widely use *C. elegans* for modeling hypoxia to decipher UPRmt. Although UPRmt has been implicated in hypoxic adaptation in several models, whether it is consistently protective, detrimental, or context-dependent remains unclear, particularly in human disease. The knowledge gaps regarding molecular triggers under hypoxia, temporal dynamics, tissue specificity, UPRmt versus ISR contributions, differences between acute and chronic hypoxia, effects of reoxygenation, etc., will aid our comprehension of the UPRmt response under hypoxic conditions. Hence, further research is needed to fully explore the link between hypoxia and UPRmt, especially in human phenotypes.
